# Association of MTHFD1 G1958A Polymorphism with Gestational Diabetes Mellitus

**DOI:** 10.7759/cureus.53287

**Published:** 2024-01-31

**Authors:** Papa Kusuma Bunga, Vijaya Sirisha Balaga, Riya Raju, Tarun Kumar Suvvari, Nagarjuna Sivaraj, Gaurang Narayan, Rithika Ramadugu, Nithya Arigapudi, Mahesh Babu Kande, Arun Panchanani

**Affiliations:** 1 Research and Development, Great Eastern Medical School & Hospital, Srikakulam, IND; 2 Obstetrics and Gynaecology, Great Eastern Medical School & Hospital, Srikakulam, IND; 3 Internal Medicine, Maharajah Institute of Medical Sciences, Vizianagaram, IND; 4 General Medicine, Rangaraya Medical College, Kakinada, IND; 5 Research, Squad Medicine and Research (SMR), Visakhapatnam, IND; 6 Obstetrics and Gynecology, Indira Gandhi Government Medical College & Hospital, Nagpur, IND; 7 Surgery, Kamineni Academy of Medical Science And Research Centre, Hyderabad, IND; 8 Genetics, Dr. Pinnamaneni Siddhartha Institute of Medical Sciences & Research Foundation, Vijayawada, IND; 9 Internal Medicine, Great Eastern Medical School & Hospital, Srikakulam, IND

**Keywords:** case-control study, gene polymorphism, gdm, gestational diabetes mellitus, mthfd1 g1958a polymorphism

## Abstract

Background

The MTHFD1 G1958A polymorphism is a common variation in the gene encoding methylenetetrahydrofolate dehydrogenase 1 (MTHFD1), an enzyme crucial for folate metabolism. This study investigated the association between the MTHFD1 G1958A polymorphism, which is involved in folate metabolism, and gestational diabetes mellitus (GDM) risk.

Methods

A case-control study was conducted and 304 pregnant women (152 with gestational diabetes as cases and 152 healthy pregnant as controls) participated in the study. The polymerase chain reaction-restriction fragment length polymorphisms (PCR-RFLP) techniques were used to determine the MTHFD1 1958G>A polymorphism genotypes.

Results

Analysis of genotype frequencies revealed a statistically significant difference (p-value < 0.05) between the GDM group and the control group, suggesting a potential association between this gene variant and the development of GDM. Interestingly, while allele frequencies alone did not show a significant association with GDM risk, analysis in a recessive model (both severe and mild forms) demonstrated a strong link between the homozygous AA genotype and increased susceptibility to GDM.

Conclusion

This study provides the first evidence linking the MTHFD1 G1958A polymorphism and GDM risk in an Indian setting. These findings warrant further investigation into the functional impact of the MTHFD1 G1958A polymorphism and its potential role in the pathogenesis of GDM.

## Introduction

*MTHFD1* gene produces methylenetetrahydrofolate dehydrogenase 1 (MTHFD1), which is a hinge enzyme in the metabolism of folic acid. MTHFD1 enzyme catalyzes three reversible and sequential processes in the pathway of tetrahydrofuran (THF) conversion. As a result, converted folate substrates are involved in several pathways leading to nucleotide formation and DNA methylation [1]. The MTHFD1 enzyme plays a crucial role in indirectly supplying one-carbon units for methylation reactions during the synthesis of 5,10-methylene-THF. Additionally, the *MTHFD1* gene is responsible for the production of 10-formyl-THF, which is essential for DNA synthesis. A polymorphism at nucleotide 1958 G>A leads to the substitution of alanine for glycine at codon 653, located in the domain 10-formyl-THF synthase of the enzyme. In cases of continuous limitation of folate availability, uncontrolled repair cycles can result in frequent breaks in the DNA molecule and chromosome damage [2]. So, if the DNA molecule breaks, which can lead to neural tube defects (NTDs), different types of syndromes can form in embryonic development during pregnancy [3]. *MTHFD1* gene polymorphism is mainly associated with placental influence on recurrent pregnancy loss, congenital heart diseases in early infants, intrauterine growth restriction, preeclampsia, placental abruption, and fetal death [4-8].

In recent times, there has been an increasing prevalence of gestational diabetes mellitus (GDM). GDM is defined as glucose intolerance that occurs or is first detected in pregnancy. GDM is the main cause of mortality and morbidity for both mother and child [9,10]. A 70% chance of getting diabetes within 28 years after delivery exists for gravidas with diabetes mellitus [11]. Studies conducted globally have demonstrated that mothers of diverse racial backgrounds and geographical locations have different risk factors for developing GDM [11-14]. Several studies were focused on the different types of gene polymorphisms in elevated folate concentrations during pregnancy, which can also lead to GDM. Based on the elevated folate levels, some studies mainly focused on the association between *MTHFR C677T* gene polymorphism and GDM [15-17].

Thus, the main objective of this study was to investigate *MTHFD1 G1958A* polymorphism and risk for GDM.

## Materials and methods

A case-control study was conducted from March 2021 March to December 2022 in a tertiary care teaching hospital, Great Eastern Medical School & Hospital, Srikakulam. The Institutional Ethical Committee of Great Eastern Medical School & Hospital, Srikakulam approved the study (approval number: 06A/IEC/GEMS&H/2022). Informed consent was taken from all patients after explaining the purpose of the study.

Primi and multigravida pregnant women with gestational age ≥ 24 weeks, aged 18-45, with GDM diagnosed by the criteria made by the International Association of Diabetes in Pregnancy Study Group (IADPSG) were included in the study. Exclusion criteria include participants with pre-existing diabetes (type 1 or type 2), multiple gestations, preeclampsia, eclampsia, hydatidiform mole, intrauterine growth restriction (IUGR), chronic hypertension, pregnancy of fewer than 24 weeks, hypothyroid pregnancy, psychological problems, heart problems, pre-existing coagulation disorders, severe renal or liver disease, infections, and systemic illness, and unwillingness to comply. Patients who had a diabetes condition during pregnancy, i.e., GDM, were considered as cases, and patients who did not have diabetes and other related complications were considered as controls. 

Stratified systemic sampling was used and 304 pregnant women (152 Cases and 152 Controls) in the age group of 25-35 years were recruited as study participants during the study period. All of the subjects enrolled in this study received a daily dose of 1 mg of folic acid.

Baseline parameters

The data of baseline parameters like age group (25-35 years), previous obstetrics history, type of conceiving pregnancy (normal or with complications), gestational age, and blood groups were collected for basic analysis of disease confirmation.

Collection of sample

A quantity of 3 ml venous blood was taken in ethylenediaminetetraacetic acid (EDTA) containers from selected subjects. The collected blood samples were stored at -40^o^C until processing. Through the salting out procedure, genomic DNA was isolated from the collected blood samples and stored at -20^o^C for further analysis [18]. Due to the reduced folic acid supplementation during pregnancy, this may cause genetic malformations in folate metabolism [16,19]. So, as evidence, we had to choose the single loci of *MTHFD1 G1958A* for our investigation.

Molecular analysis

The polymerase chain reaction-restriction fragment length polymorphism (PCR-RFLP) technique was used to determine the *MTHFD1 1958G>A* polymorphism genotypes.

PCR Amplification

Sense: 5’-CACTCCAGTGTTTGTCCATG-3’ and Antisense: 5’-GCATCTTGAGAGCCCTGAC-3’ primers were used in PCR amplification for analyzing the gene mutations in the *MTHFD1 G1958A* gene. The amplification was performed in 15 µl total volume with 8 µl of DH_2_O, 4 µl of Taq mix, 1 µl of forward primer, 1 µl of reverse primer, and 1 µl of isolated DNA (template DNA). The 15 µl reaction was obtained by the initial denaturation, five minutes at 94^o^C, denaturation for one minute at 94^o^C, annealing for one minute at 52.5^o^C, extension for one minute at 72°C, and final extension at 72°C for five minutes.

RFLP Analysis

The 331 bp amplificated product was digested with MspI enzyme for three hours at 37°C (98°F). The digested product was determined by the gel electrophoresis to identify the restricted site. As a result, fragments of 166bp and 70bp were produced at the present G allele and 266bp fragment was also produced when the A allele was present.

The dominant and recessive model of gene polymorphism describes the inheritance patterns of genetic variations within a population. In this context, a gene polymorphism refers to the presence of different versions (alleles) of a gene in the same population. The dominant-recessive model is a classic way to understand how these alleles interact and determine an individual's phenotype.

Statistical analysis

The OpenEpi web tool was used to calculate the genotype and allele frequencies of *MTHFD1 1958G>A* gene polymorphism with the help of the chi-square test (Hardy-Weinberg Equilibrium chi-square test). P-value <0.05 was considered as statistically significant. Student T-test was performed to calculate the baseline parameters such as age group (25-35 years), previous obstetric history, type of conceiving pregnancy (normal or pregnancy with complications), gestational age and blood groups. The sample size was determined using an online calculator tool, resulting in a total sample size of 304. This sample was divided into two groups, with 152 cases and 152 controls. The confidence level was set at 99%, indicating a 99% certainty that the true value falls within a range of ±5%. The population proportion used for this calculation was 15% of the prevalence.

## Results

Table 1 shows that there is a statistically significant difference between baseline parameters of GDM cases and controls. The parameters of age and normal pregnancy showed significant association with GDM cases when compared with controls (p=0.000 and p=0.000, respectively). Most participants suffered from GDM in the condition of high-risk pregnancy, hence the condition of high-risk pregnancy was a major factor for increasing GDM. Obstetric history, gestational age, and blood groups did not reveal any statistical significance difference between the two groups (p=0.251, p=0.121, and p=0.292, respectively).

**Table 1:** Baseline Characteristics of Cases and Controls (n=304)

Baseline characteristics	Cases with GDM (n=152)	Controls (n=152)	Chi-square	P-value
Age (years)				
>25	70 (46.05%)	110 (72.36%)	23.79	0.000
25-30	58 (38.15%)	31 (20.39%)		
30-35	27 (17.76%)	11 (7.23%)		
Obstetric history				
Primigravida	78 (51.31%)	68 (44.73%)	1.318	0.251
Multigravida	74 (48.68%)	84 (55.26%)		
Normal pregnancy				
Yes	51 (33.55%)	118 (77.63%)	59.81	0.000
No	101 (66.44%)	34(66.44%)		
Gestational age (weeks)				
>35	62 (40.78%)	49 (32.23%)	2.398	0.121
<35	90 (59.21%)	103 (67.76%)		
Blood group				
A+	22 (14.47%)	28 (18.42%)	7.319	0.292
B+	32 (21.05%)	46 (30.26%)		
AB+	79 (51.97%)	59 (38.81%)		
O+	07 (4.60%)	06 (3.94%)		
A-	03 (1.97%)	05 (3.28%)		
B-	04 (2.63%)	05 (3.28%)		
O-	05 (3.28%)	03 (1.97%)		

Table 2 represents the genotype and allele frequencies of *MTHFD1* gene polymorphisms between both groups. Genotypes of GG and AA had higher frequency in the Control group than in the GDM group and the genotype of GA was higher in the GDM group than in the Control group. There was a statistically significant difference between the two groups (p=0.005). The G allele showed a higher frequency in the Control group and the A allele showed a higher frequency in the GDM group. Allele frequency does not show any association with GDM risk (O=0.8164; 95%CI=0.5836-1.142, and p-value=0.124).

**Table 2:** Genotype and Allele Frequencies of MTHFD1 (1958G>A) Gene Polymorphisms in Cases and Controls. GDM: gestational diabetes mellitus

Genotype	GDM group (N=152)		Non-GDM group (N=152)		Chi-square	P-value
	Frequency	Percentage	Frequency	Percentage		
GG	52	34.21	72	47.36	10.37	0.005
GA	58	38.15	33	21.71		
AA	42	27.63	47	30.92		
Allele	-	-	-	-	OR (95% CI)	P-value
G	169	55.59	184	60.52	0.8164 (0.5836-1.142)	0.124
A	135	44.40	120	39.47		

Table 3 presents the association between maternal diabetes mellitus and *MTHFD1* gene variants. In the dominant model, there was no association difference between overall (OR=0.853; 95%CI=0.5201-1.441; p=0.307), severe (OR=1.019; 95%CI=0.5455-1.881; p-0.531), and mild forms (OR=0.6744; 95%CI=0.3257-1.351; p=0.154). Importantly, the recessive model reveals a high risk in the mild form in GDM cases (OR=0.3354; 95%CI=0.1646-0.6619: p=0.000). Overall (OR=0.5778; 95%CI=0.3542-09412: p=0.031) and severe (OR=0.5068; 95%CI=0.2761-0.9201; p=0.011) forms in the recessive model showed a significant association to GDM risk. In the allele, variants showed insignificant association in overall (OR=1.225; 95%CI=0.8757-1.714; p=0.124), severe (OR=0.796; 95%CI=0.5211-1.211; p=0.155) and mild (OR=1.297; 95%CI=0.8512-1.974; p=0.120) forms. 

**Table 3:** Association Between GDM and MTHFD1 Gene Variants GDM: gestational diabetes mellitus

Genetic Model	Overall			Severe GDM			Mild GDM		
Dominant model GG + GA Vs. AA	OR	95% CI	P-value	OR	95% CI	P-value	OR	95% CI	P-value
	0.853	0.5201-1.441	0.307	1.019	0.5455-1.881	0.531	0.6744	0.3257-1.351	0.154
Recessive model AA + GA Vs. GG	0.5778	0.3542-09412	0.031	0.5068	0.2761-0.9201	0.011	0.3354	0.1646-0.6619	0.000
Allele G Vs. A	1.225	0.8757-1.714	0.124	0.796	0.5211-1.211	0.155	1.297	0.8512-1.974	0.120

## Discussion

The gene, which encodes the MTHFD1 enzyme, is located on the long arm of chromosome 14 (14q 24). MTHFD1 is a trifunctional enzyme and is a maternal risk for severe placental abruption [19]. MTHFD1 has catalytic activity, which is indirectly involved in purines, and pyrimidine synthesis is mainly required for de novo DNA synthesis for embryonic development [19]. Previous studies were focused on *MTHFD1* gene polymorphism associated with various diseases like an elevated risk of breast cancer, colorectal cancer, and methotrexate sensitivity in ALL [19-22]. G1958A is a common gene polymorphism in the *MTHFD1* gene, and plays an essential role in folate metabolism. This gene polymorphism can be associated with cancer due to the various consequences of cell control and various alterations that occur during DNA synthesis [23]. In recent years, there has been an increasing prevalence of pregnant women suffering from GDM, which affects around 5-10% of pregnancies worldwide [24].

Several studies demonstrated that older maternal age, previous obstetric history (e.g., macrosomia, stillbirth, abortion, premature delivery, congenital anomaly, primigravida, family history of diabetes, blood group, and gestational age were also risk factors for the development of GDM [25-28]. However, in our study, insignificant chi square p-values were observed in obstetric history and gestational age with GDM (P-values 0.251 and 0.121, respectively). Few studies reported that maternal age increases linearly with GDM risk [29,30]. Few studies reported that females who were pregnant at the age of 30-34 were more prone to develop GDM [31,32]. In the current study, it was found that maternal age is also an independent risk factor for the development of maternal diabetes and this was statistically significant (P-value=0.000).

The antigens of ABO blood groups are expressed by various human cells and organs, and they have been implicated in systemic diseases such as GDM [33,34]. Several studies conducted in different countries have explored the relationship between maternal diabetes and ABO blood groups, with findings suggesting correlations between different ABO blood groups and the development of maternal diabetes during various trimesters of pregnancy [35-37]. A retrospective case-control study demonstrated that the blood group AB was identified as a risk factor for GDM [36]. However, the present study revealed that ABO blood groups did not exhibit a significant association with the development of maternal diabetes, and was found to be insignificant. This suggests that further research is necessary to elucidate the potential role of ABO blood groups in the development of maternal diabetes.

Parle-McDermott et al. conducted the first investigation into the relationship between pregnancy loss and the *MTHFD1 G1958A* gene polymorphism [38]. Their research revealed that the *MTHFD1 *gene polymorphism serves as an independent predictor of pregnancy loss in the second trimester. While the phenotypic effect of the *MTHFD1 *gene variant is not currently known, it is postulated to potentially influence the rate of DNA synthesis and, consequently, the rate of cell division, which is critical during pregnancy and fetal development. These effects may also have implications for the development of GDM. Our study was the first to identify *MTHFD1 G1958A* gene polymorphisms in GDM in an Indian setting. Although we lacked direct evidence linking GDM to the *MTHFD1 G1958A *gene polymorphism, we had general evidence of its association with the gene.

A cohort study done by Jankovic-Karasoulos et.al. stated that dietary information, folic acid supplementation, and maternal and paternal genotypes were identified as independent risk factors for pregnancy complications [39]. There was a correlation observed between maternal genotypes and an elevated likelihood of developing gestational hypertension, preeclampsia, GDM, small for gestational age, and spontaneous preterm birth. However, the current study found that ī polymorphism had an association with GDM in the Indian population, which might be due to environmental effects or lack of awareness of proper dietary intake during pregnancy.

In the present study, we found a significant relationship between the genotypes of the *MTHFD1 G1958A* gene polymorphism and GDM (p=0.005). Specifically, the G allele exhibited a higher frequency in the control group, while the A allele showed a higher frequency in the cases. However, allele frequency did not appear to be associated with the risk of maternal diabetes (OR=0.8164; 95%CI=0.5836, 1.142; p-value=0.124).

Importantly, the present study focused on the subsequent interactions between gene variants of the *MTHFD1* gene polymorphism and GDM. It revealed that the recessive model showed a high risk in mild-form GDM cases (OR=0.3354; 95%CI=0.1646-0.6619; p=0.000). Overall, the recessive model showed a significant association with the risk of GDM in both mild (OR=0.5778; 95%CI=0.3542-0.9412; p=0.031) and severe (OR=0.5068; 95%CI=0.2761-0.9201; p=0.011) forms. These findings underscore the potential significance of the *MTHFD1* gene polymorphism in the risk of GDM, particularly in the recessive model.

The AA genotype of the *MTHFD1 G1958A *polymorphism shows promise as a GDM risk biomarker in the Indian population, potentially enabling early detection and targeted interventions. However, its utility as a standalone biomarker is limited. Future studies incorporating diverse populations, gene-environment interactions, and folate levels are crucial to refine its predictive power and pave the way for its potential integration into personalized GDM prevention strategies while considering cost-effectiveness and clinical feasibility. 

The present study provides valuable insights into the potential contribution of *MTHFD1 G1958A* to GDM susceptibility, but there are a few limitations which require further investigation. The sample size is small and may limit the generalizability of the findings. Additionally, the study's focus on an Indian setting may restrict the broader applicability of the results to other populations with different genetic backgrounds, ethnicities, and environmental influences. Moreover, the study's findings are based on associations and do not establish causality, warranting further functional studies to elucidate the mechanistic implications of the *MTHFD1 G1958A* polymorphism in the development of GDM. Future studies with larger, diverse populations and comprehensive analyses incorporating gene-environment interactions are crucial to solidify the understanding of this association and its clinical implications.

## Conclusions

Our study focused on the subsequent interactions between gene variants of *MTHFD1* gene polymorphism and GDM. Significant associations were identified between age and pregnancy with GDM. The prevalence of GDM is notably higher in high-risk pregnancies, indicating that high-risk pregnancy is a major contributing factor to the increased incidence of GDM. The findings did not reveal any statistical significance for obstetric history, gestational age, and ABO blood groups in relation to GDM.

This study is the first to provide evidence linking the *MTHFD1 G1958A* polymorphism and GDM risk in an Indian setting. We reported an association between the *MTHFD1* gene variants in the recessive model and GDM. These findings warrant further investigation into the functional impact of the *MTHFD1 G1958A* polymorphism and its potential role in the pathogenesis of GDM.
